# A Treatise on the Structure, Economy, and Diseases of the Ear; Being the Essay for Which the Fothergillian Gold Medal Was Awarded by the Medico-Society of London

**Published:** 1843-04-01

**Authors:** 


*A Treatise on the Structure, Economy, and Diseases of the Ear; being the Essay for which the Fothergillian gold medal was awarded by the Medico-Society of London.* By George Pilcher, Late Lecturer on Anatomy, &c. &c. First American, from the second London Edition. With Notes. Philadelphia, 1843. Barrington & Haswell. 8vo. pp. 299.

The original of the present Treatise obtained the Fothergillian Medal, and its subsequent publication was owing to the advice of the friends of the author; that their advice was not injudicious is proved by the favour it has already received, having reached in a short time a second edition.

Mr. Pilcher thinks that

"It might have been reasonably anticipated that the immense improvements which have been introduced into Ophthalmic Surgery by the labours of some of the most distinguished members of our profession, would have induced the educated practitioner to investigate those numerous diseases to which the ear is obnoxious. Such, unhappily for the welfare of mankind, has not been the result; and thus it happens that even at the present time, in this country at least, Aural Surgery is either almost entirely neglected, or for the most part is left in the hands of the ignorant empiric. In consequence, therefore, of what must be considered a dereliction of duty on the part of English surgeons, the unfortunate sufferers from these distressing maladies are, in many instances, abandoned to their fate, or compelled to seek relief from the employment of nostrums which it would be but too charitable to regard as being merely harmless in their operation."

Until the diseases of the ear be subjected to general pathological principles in their treatment, and until they become part of the regular education of the physician, we agree with our author in thinking that it will "be in vain to hope for any considerable extension of the very limited knowledge which is at present possessed on this interesting class of diseases."

After some general observations on the sense and on the comparative development of the organ, the anatomy and the physiology of the ear are discussed in a good, condensed style. The subject of acoustics is touched upon as introductory to the function of hearing.

Part Second comprises the Abnormal Condition of the Ear; this includes the Development and Malformation of the Ear, and its several parts, and Deaf-Dumbness. The latter section is very interesting. With regard to the greater frequency of deaf-dumbness in males, our author observes,

"It is a curious circumstance that deaf-dumbness is much more frequent in males than in females, which is in violation of the ordinary law, that as the power of formation is weaker in the female fœtus than in the male, so are the deficiencies more frequent. Mr. May, the director of the deaf and dumb school at Vienna, stated that the proportion of deaf boys to girls was as four to one."

As respects its hereditary character he says:

"Congenital deafness does not appear to be hereditary, as most of the parents of deaf children have had no defect of their organs; and it is a rare circumstance to meet a case of a deaf child who was the fruit of parents, either one or both similarly effected. On the other hand, several children of one family will be thus defective, without any known cause, while the others will be perfectly healthy. Kramer relates the singular instance of 'a man and his wife, of the name of Hartness, both of them healthy, and having no hereditary predisposition to any disease of the Ear in their family on either side, who have five daughters and six sons; the latter were all born deaf-dumb, whilst the daughters, without exception, heard perfectly well. The mother of these eleven children is not aware of any circumstance that distinguished her pregnancies from each ether, though the children are so remarkably differently endowed. She was always healthy and active. One of their children has married a deaf-dumb girl, but their marriage has been childless.' A healthy couple, residing in the parish of Bishopsgate, with a large family, have two of their daughters deaf and dumb; the eldest, about forty of years age, has married a *sourd-muet*, and become the mother of several children, all of whom enjoy perfect audition; the youngest, aged twenty-six, can hear a few sounds, and indistinctly utter a few words, which, however, appear to be simply vocalized. The roof of her mouth is very concave; the right membrana tympani is abnormally oblique and very transparent, the left is opaque, but natural in shape and size,—the Eustachian tubes are healthy,—it is probable that the diameter of the right tympanum is smaller than usual, and that malformation exists in both labyrinths."

The Third Part treats of the Diseases of the Ear.

Our author seems practically familiar with his subject. His descriptions are well written, and his pathological views and therapeutics are, in general, sound.

The work is handsomely illustrated by sixteen well-executed lithographs.

